# Supplementary material for: NCDB Analysis of Melanoma 2004–2015: Epidemiology and Outcomes by Subtype, Sociodemographic Factors Impacting Clinical Presentation, and Real-World Survival Benefit of Immunotherapy Approval
Source: Cancers (Basel). 2021 Mar 22;13(6):1455. doi: 10.3390/cancers13061455 (PMC8004999; doi:10.3390/cancers13061455)
Supplement: Supplementary file 1 [file cancers-13-01455-s001.pdf]

cancers

MDPI

Supplementary Material

Table S1. ICD-O-3 codes used in the study.

Histology/Site	International Classification of Diseases for Oncology, third edition (ICD-O-3)
	Histology codes
**Melanoma**	8720 8744 8730 8745 8722 8770 8771 8742 8761 8740 8741 8723 9044 8746 8721 8772 8773 8774 8743 8728
**Cutaneous Melanoma**	8720 8744 8730 8745 8722 8770 8771 8742 8761 8740 8741 8723 8721 8772 8773 8774 8743
	Site Codes
**Skin**	C44
**Genital (Male)**	C600 C609 C632 C601 C602 C608 C619 C621 C629 C631
**Genital (Female)**	C510 C511 C512 C513 C518 C519 C529 C530 C531 C538 C539 C540 C541 C549 C559 C569 C578 C579
**Urinary**	C649 C659 C669 C671 C672 C673 C674 C675 C678 C679 C680 C688 C689
**Gastrointestinal Tract**	C151 C153 C154 C155 C158 C159 C160 C161 C162 C163 C165 C166 C168 C169 C170 C171 C172 C178 C179 C180 C182 C186 C187 C189 C199 C209 C210 C211 C212 C218
**Head and Neck**	C000 C001 C002 C006 C003 C004 C005 C009 C019 C020 C021 C022 C023 C028 C029 C030 C031 C039 C040 C049 C050 C051 C052 C058 C059 C060 C061 C062 C068 C069 C079 C080 C081 C089 C090 C091 C099 C100 C102 C108 C109 C110 C111 C112 C113 C118 C119 C129 C139 C140 C148 C320 C321 C322 C329 C300 C301 C310 C311 C312 C313 C318 C319
**Ocular**	C690 C693 C694 C691 C692 C695 C696 C698 C699

Table S2a. AJCC clinical stage for cutaneous melanoma (This includes AJCC 6th and 7th edition for the respective years).

**AJCC Clinical Stage**	***n* = 262,872**	%
1	177,121	67.38
2	52,185	19.85
3	14,069	5.35
4	19,497	7.42

Table S2b. Differences in treatment facility type by patients' race, income and education level.

			**Community cancer program**	**Academic/research program**	**Integrated Network Cancer Program**	**Chi Squared Test *P* Value**
**Race**	**Caucasians**	*n*	8,858	7,469	2,486	<0.001
		%	47.08	39.7	13.21	
	**African Americans**	*n*	122	212	55	
		%	31.36	54.5	14.14	
	**Others**	*n*	133	207	64	
		%	32.92	51.24	15.84	
	**Total**	***n***	**9,113**	**7,888**	**2,605**	
**Socio Economic Status:** *Median household income for patient's zip code*	**<40,227 (level 1)**	*n*	747	609	129	<0.001
		%	50.3	41.01	8.69	
	**40,227–50,353 (level 2)**	*n*	1,761	1,253	349	
		%	52.36	37.26	10.38	
	**50,354–63332 (level 3)**	*n*	2,744	2,039	748	
		%	49.61	36.86	13.52	
	**>46,000 to >63,333 (level 4)**	*n*	3,816	3,966	1,369	
		%	41.7	43.34	14.96	
	**Total**	***n***	**9,068**	**7,867**	**2,595**	
**Education Level:** *Number of adults in the patient's zip code who did not graduate from high school*	**> 29% (level 1)**	*n*	946	815	177	<0.001
		%	48.81	42.05	9.13	
	**20–28.9% (level 2)**	*n*	2,045	1,568	493	
		%	49.81	38.19	12.01	
	**14–18.9% (level 3)**	*n*	2,428	2,022	719	
		%	46.97	39.12	13.91	
	**< 14% (level 4)**	*n*	3,654	3,464	1,209	
		%	43.88	41.6	14.52	
	**Total**	***n***	**9,073**	**7,869**	**2,598**	

Table S3. Burden of invasive melanoma between 2004 and 2015.

**Invasive Melanoma**	**Cutaneous**			**Ocular**			**Mucosal**		
**Year**	*Total (n)*	*Metastatic (n)*	*Metastatic (% of total)*	*Total (n)*	*Metastatic (n)*	*Metastatic (% of total)*	*Total (n)*	*Metastatic (n)*	*Metastatic (% of total)*
**2004**	27042	1249	4.84	1240	26	2.14	535	58	12.16
**2005**	29168	1285	4.61	1331	20	1.53	587	55	10.34
**2006**	29664	1365	4.82	1555	23	1.50	544	51	10.34
**2007**	30945	1476	5.01	1619	24	1.50	631	73	13.08
**2008**	32259	1605	5.24	1611	30	1.90	627	56	9.81
**2009**	33723	1679	5.24	1329	24	1.84	751	89	13.44
**2010**	34426	1821	5.59	1390	22	1.61	709	66	10.26
**2011**	35575	1714	5.06	1455	29	2.03	730	76	11.62
**2012**	36647	1624	4.64	1525	25	1.67	721	78	12.13
**2013**	39265	1815	4.85	1648	28	1.73	787	88	12.59
**2014**	40979	1857	4.75	1609	44	2.81	835	82	10.89
**2015**	43549	2002	4.82	1727	31	1.83	851	101	13.47

Table S4a. All-cause mortality for invasive melanoma diagnosed between years 2004–2015.

Invasive Melanoma (2004–2015)	Non- Metastatic *n* (%)	Metastatic *n* (%)	Chi Squared *P* Value
**Cutaneous**	80,760 (22.31%)	15,601 (80.04%)	<0.001
**Ocular**	4999 (29.67%)	278 (85.28%)	<0.001
**Mucosal**	3765 (62.25%)	764 (87.51%)	<0.001

Table S4b. All-cause mortality for cutaneous melanoma by histology and extent of disease diagnosed between years 2004–2015.

	Histology	Died		Totals	
		No	Yes		
**Metastatic Cutaneous Melanoma**	**Melanoma NOS (*n*)**	**3,238**	**13,294**	16,532	Chi Squared *P* Value <0.001
	%	19.59	80.41		
	**Acral Lentiginous (*n*)**	**14**	**83**	97	
	%	14.43	85.57		
	**Nodular (*n*)**	**280**	**1,216**	1,496	
	%	18.72	81.28		
	**Superficial Spreading (*n*)**	**127**	**353**	480	
	%	26.46	73.54		
	**Lentigo Maligna (*n*)**	**13**	**53**	66	
	%	19.7	80.3		
	**Others (*n*)**	**219**	**602**	821	
	%	26.67	73.33		
	**Histology**	**Died**			
		**No**	**Yes**		
**Non-metastatic Cutaneous Melanoma**	**Melanoma NOS (*n*)**	**133,866**	**38,400**	172,266	Chi Squared *P* Value <0.001
	%	77.71	22.29		
	**Acral Lentiginous (*n*)**	**3,353**	**1,618**	4,971	
	%	67.45	32.55		
	**Nodular (*n*)**	**20,899**	**13,370**	34,269	
	%	60.99	39.01		
	**Superficial Spreading (*n*)**	**97,360**	**17,545**	114,905	
	%	84.73	15.27		
	**Lentigo Maligna (*n*)**	**14,706**	**5,022**	19,728	
	%	74.54	25.46		
	**Others (*n*)**	**11,067**	**4,805**	15,872	
	%	69.73	30.27		

Table S5. Impact of treatment facility type on the odds of mortality (Multivariate analysis).

Factor	OR	95% CI	*P* value
**Treating Facility Type**			
Academic/research program vs. Community cancer program	0.75	0.69–0.81	<0.001
Integrated Network Cancer Program vs. Community cancer program	0.92	0.83–1.04	0.216
**Female vs. Male**	0.88	0.82–0.95	0.002
**Age**	1.02	1.01–1.02	<0.001
**Insurance Status**			
Private Insurance vs. Not insured	0.62	0.52–0.76	<0.001
Medicaid vs. Not insured	0.93	0.73–1.18	0.558
Medicare or other government insurance vs. Not insured	0.69	0.57–0.85	<0.001
**Charlson Deyo score**			
1 vs. 0	1.3	1.17–1.45	<0.001
2 vs. 0	1.66	1.36–2.02	<0.001
3 vs. 0	2.84	2.04–3.93	<0.001
**Number of adults in the patient's zip code who did not graduate from high school**			
20–28.9% vs. > 29%	0.98	0.84–1.13	0.764
14–18.9% vs. > 29%	0.98	0.84–1.15	0.812
< 14% vs. > 29%	0.95	0.81–1.12	0.574
**Socio Economic Status:** ***Median household income for patient's zip code***			
40,227–50,353 (level 2) vs. <40,227 (level 1)	0.95	0.79–1.13	0.543
50,354–63332 (level 3) vs. <40,227 (level 1)	0.91	0.76–1.07	0.263
>46,000 to >63,333 (level 4) vs. <40,227 (level 1)	0.76	064–0.91	0.003
**Invasive melanoma subtype**			
Ocular vs. Cutaneous	1.61	1.16–2.24	0.004
Mucosal vs. Cutaneous	1.78	1.44–2.20	<0.001
**Geographical location**			
West vs. North East	1.01	0.89–1.13	0.911
Midwest vs. North East	1.26	1.12–1.42	<0.001
South vs. North East	0.9	0.81–1.00	0.051

Table S6. Adjusted HR for survival with metastatic melanoma post Ipilimumab approval (2011–2015) versus prior to Ipilimumab approval (2004–2010).

Factors	HR	95% CI	P value
**Year of diagnosis (2011–2015 vs. 2004–2010)**	0.81	0.78–0.83	<0.001
**Age**	1.01	1.01–1.01	<0.001
**Female vs. Male**	0.92	0.89–0.95	<0.001
**Race**			
African Americans vs. Caucasians	1.08	0.96–1.21	0.199
Others vs. Caucasians	1.01	0.91–1.13	0.801
**Charlson Deyo score**			
1 vs. 0	1.19	1.14–1.24	<0.001
2 vs. 0	1.44	1.34–1.54	<0.001
3 vs. 0	1.85	1.69–2.03	<0.001
**Treating Facility Type**			
Academic/research program vs. Community cancer program	0.83	0.8–0.86	<0.001
Integrated Network Cancer Program vs. Community cancer program	0.98	0.93–1.03	0.372
**Insurance Status**			
Private Insurance vs. Not insured	0.66	0.61–0.71	<0.001
Medicaid vs. Not insured	0.95	0.86–1.05	0.293
Medicare or other government insurance vs. Not insured	0.72	0.66–0.78	<0.001
**Socio Economic Status:** ***Median household income for patient's zip code***			
40,227–50,353 (level 2) vs. <40,227 (level 1)	0.98	0.91–1.05	0.572
50,354–63332 (level 3) vs. <40,227 (level 1)	0.97	0.9–1.04	0.368
>46,000 to >63,333 (level 4) vs. <40,227 (level 1)	0.9	0.84–0.97	0.005
**Number of adults in the patient's zip code who did not graduate from high school**			
20–28.9% vs. > 29%	0.98	0.92–1.05	0.597
14–18.9% > 29%	1.01	0.94–1.08	0.802
<14% > 29%	0.97	0.91–1.04	0.42
**Geographical location**			
West vs. North East	0.99	0.94–1.05	0.822
Midwest vs. North East	1.07	1.02–1.12	0.007
South vs. North East	0.98	0.93–1.02	0.319

Table S7. Adjusted HR for survival of Caucasians with metastatic melanoma post Ipilimumab approval (2011–2015) versus prior to Ipilimumab approval (2004–2010).

Factors	HR	95% CI	*P* value
**Year of diagnosis** (2011–2015 vs. 2004–2010)	0.80	0.78–0.83	<0.001
**Female vs. Male**	0.92	0.89–0.95	<0.001
**Age**	1.01	1.009–1.01	<0.001
**Treating Facility Type**			
Academic/research program vs. Community cancer program	0.83	0.8–0.86	<0.001
Integrated Network Cancer Program vs. Community cancer program	0.98	0.93–1.03	0.365
**Insurance Status**			
Private Insurance vs. Not insured	0.64	0.59–0.69	<0.001
Medicaid vs. Not insured	0.95	0.86–1.05	0.297
Medicare or other government insurance vs. Not insured	0.70	0.64–0.76	<0.001
**Socio Economic Status:** ***Median household income for patient's zip code***			
40,227–50,353 (level 2) vs. <40,227 (level 1)	0.99	0.92–1.06	0.703
50,354–63332 (level 3) vs. <40,227 (level 1)	0.98	0.91–1.05	0.505
>46,000 to >63,333 (level 4) vs. <40,227 (level 1)	0.90	0.84–0.98	0.009
**Geographical location**			
West vs. North East	0.98	0.93–1.03	0.378
Midwest vs. North East	1.06	1.01–1.11	0.02
South vs. North East	0.96	0.92–1.01	0.083
**Charlson Deyo score**			
1 vs. 0	1.19	1.14–1.25	<0.001
2 vs. 0	1.43	1.33–1.54	<0.001
3 vs. 0	1.84	1.68–2.02	<0.001
**Number of adults in the patient's zip code who did not graduate from high school**			
20–28.9% vs. > 29%	0.97	0.91–1.03	0.343
14–18.9% > 29%	0.99	0.92–1.05	0.683
<14% > 29%	0.96	0.89–1.03	0.238

Table S8. Adjusted HR for survival of African Americans with metastatic melanoma post Ipilimumab approval (2011–2015) versus prior to Ipilimumab approval (2004–2010).

Factors	HR	95% CI	*P* value
**Year of diagnosis** (2011–2015 vs. 2004–2010)	0.80	0.62–1.02	0.073
**Female vs. Male**	0.70	0.55–0.88	0.003
**Age**	1.01	0.99–1.02	0.053
**Treating Facility Type**			
Academic/research program vs. Community cancer program	0.98	0.75–1.29	0.903
Integrated Network Cancer Program vs. Community cancer program	1.13	0.78–1.63	0.526
**Insurance Status**			
Private Insurance vs. Not insured	1.03	0.65–1.63	0.89
Medicaid vs. Not insured	1.09	0.63–1.9	0.757
Medicare or other government insurance vs. Not insured	1.18	0.75–1.87	0.481
**Socio Economic Status:** ***Median household income for patient's zip code***			
40,227–50,353 (level 2) vs. <40,227 (level 1)	0.91	0.63–1.31	0.612
50,354–63332 (level 3) vs. <40,227 (level 1)	0.79	0.53–1.17	0.235
>46,000 to >63,333 (level 4) vs. <40,227 (level 1)	0.76	0.5–1.16	0.198
**Geographical location**			
West vs. North East	1.27	0.69–2.34	0.436
Midwest vs. North East	1.58	1.06–2.36	0.025
South vs. North East	1.47	1.06–2.02	0.019
**Charlson Deyo score**			
1 vs. 0	0.84	0.63–1.14	0.27
2 vs. 0	1.52	0.86–2.67	0.148
3 vs. 0	1.74	1.004–2.99	0.048
**Number of adults in the patient's zip code who did not graduate from high school**			
20–28.9% vs. > 29%	1.02	0.73–1.44	0.887
14–18.9% vs. > 29%	1.26	0.82–1.95	0.292
< 14% vs. > 29%	1.09	0.68–1.72	0.728

Table S9. Lymph node status and presence of metastatic disease at onset.

		Metastasis	
		**Absent**	**Present**
**Lymph Nodes**	**Negative**	159,773	1,151
	**Positive**	41,588	3,277
	**Unknown status/Missing data**	184,196	16,263
